# Serum Beta-D-Glucan in the Diagnosis of Invasive Fungal Disease in Neonates, Children and Adolescents: A Critical Analysis of Current Data

**DOI:** 10.3390/jof8121262

**Published:** 2022-11-30

**Authors:** Laura Ferreras-Antolin, Andrew Borman, Antonia Diederichs, Adilia Warris, Thomas Lehrnbecher

**Affiliations:** 1 Paediatric Infectious Diseases and Immunology Unit, St George’s University Hospitals NHS Foundation Trust, London SW17 0QT, UK; 2 Medical Research Council Centre for Medical Mycology, University of Exeter, Exeter EX4 4QD, UK; 3 UK Health Security Agency, UK National Mycology Reference Laboratory, Reference Services Division, Science Quarter, Southmead Hospital, Bristol SE1 8UG, UK; 4 Division of Paediatric Haematology and Oncology, Hospital for Children and Adolescents, University Hospital Johann Wolfgang Goethe-University, 60323 Frankfurt am Main, Germany; 5 Department of Paediatric Infectious Diseases, Great Ormond Street Hospital for Children, London WC1N 3JH, UK

**Keywords:** β-D-glucan, fungal diagnostics, neonatal invasive candidiasis, invasive fungal infections in children

## Abstract

β-D-glucan (BDG) is a cell wall component of many pathogenic fungi. The detection of BDG as an assay is clinically broadly used as a diagnostic tool. However, the current data on BDG in paediatrics are limited, prompting specific considerations about when BDG can be used in neonates and children. We aimed to analyse the available data for the use of serum BDG in neonates and immunocompromised children and adolescents; as well as to understand the extent and characteristics of the use of BDG in children in Europe.

## 1. Introduction

Invasive fungal diseases are a major cause of morbidity and mortality in selected paediatric patient populations, such as preterm neonates, children receiving therapy for cancer or those undergoing allogeneic haematopoietic stem cell transplantation (HSCT) [1,2]. Unfortunately, in these patient populations, signs and symptoms of invasive fungal diseases are often non-specific, and early diagnosis, which is associated with better outcomes, is difficult. As culture-based diagnostics lack sensitivity, are time-consuming, volume-dependent or might require invasive procedures, non-invasive diagnostic methods such as the detection of galactomannan (GM) or ß-D-glucan (BDG) and molecular tools such as polymerase chain reaction (PCR) are seen as attractive diagnostic alternatives. They may help in guiding the start and the choice of antifungal agents, as well as the duration of therapy. Non-culture-based tests are increasingly used in the clinical setting, because they offer a faster turn-around time compared to culture based diagnostic tests; they require only small sample volumes, which is of advantage particularly in neonates and younger children; and may detect infections even before they are clinically apparent [3]. Testing can be performed following three different strategies: First, samples can be evaluated on a regular basis, in order to screen high-risk patients to detect an invasive fungal disease at an early stage, prior to when clinical signs and symptoms occur. Second, the assays can be used as a diagnostic tool in patients with suspected invasive fungal diseases based on clinical or radiological findings. Finally, biomarkers can be used for monitoring the response to antifungal therapy and for the early discontinuation of empirical antifungal treatment, although most of the supporting evidence for this comes from the intensive care settings in patients with suspected invasive candidiasis [4,5].

One promising biomarker is BDG, which is a cell wall polysaccharide found in most fungi, including *Aspergillus*, *Candida*, *Fusarium* and *Pneumocystis*, but is often absent or present in limited quantities in Mucormycetes and in *Cryptococcus* [6]. Measurement of BDG levels is based on the activation by BDG of factor G of the coagulation cascade in the amoebocyte lysate from the horseshoe crab, which leads to a quantifiable transformation of a chromogenic substrate [7]. Whereas general recommendations for the use of serum BDG are conflicting [8,9], BDG testing is discouraged in neonates and immunocompromised children as data are still scarce and the optimal threshold for positivity has not been determined yet [10,11,12,13]. Although there is an increasing body of evidence on the use of BDG in paediatrics, there is significant heterogeneity among these studies. Different study designs, study populations, thresholds for positivity, variability in sampling methods or exposure to previous antifungals make results difficult to generalise.

Our review had two main objectives; (i) to analyse the available data for the use of serum BDG in neonates and immunocompromised children and adolescents; and (ii) to understand the extent and characteristics of the use of BDG in children in Europe.

## 2. Materials and Methods

### 2.1. Literature Review: Search Strategies

For the use of BDG in neonates, paediatric cancer or transplant patients, references published until 15 December 2021, and containing the search terms (beta-d—glucan or ß-d-glucan or bdg) (neonat* or child* or pediatr*) (cancer or stem cell transplantation or hsct or immunocompromised or immunodeficiency) were retrieved from the PubMed database. Publications were also manually screened for additional references. Studies with mixed adult and paediatric cases were not included in view of the different performance that BDG has in adults compared to the paediatric population and hence, the risk of skewed data.

### 2.2. Clinical Survey

A web-based survey was circulated on the 24 February 2022 to the members of ESPID (European Society of Paediatric Infectious Diseases). The survey (Supplementary Material) consisted of a maximum of eight close questions, addressing the use of BDG, rationale, population and whether or not the test was performed in-house. This was compared to the use of GM. The survey was disseminated and open to complete during February and March 2022. RedCap^TM^ (Research Electronic Data Capture, Vanderbilt University, Nashville, TB, USA) was used to collect the data. The descriptive analysis of the results was performed using STATA version 14.2. Only the results from European countries were included in the analysis and the results presented.

## 3. Results

### 3.1. Routine Use of BDG

#### 3.1.1. The Use of BDG in Preterm and Term Neonates

The use of BDG in neonates retrieved nine publications [14,15,16,17,18,19,20,21,22]. Three studies evaluated a different test than the FDA (Food and Drug Administration)-approved Fungitell^®^ [4,18,19]. The most common study design was a prospective cross-sectional and BDG was only evaluated as a diagnostic test, but not as a screening tool. A total of 597 neonates were included, most of them high risk, with 164 proven episodes of neonatal invasive candidiasis (NIC).

Only four studies calculated an optimal cut-off for positivity in premature neonates, with values ranging from 99 pg/mL to 174 pg/mL [14,15,16,17].

A recent meta-analysis estimated BDG sensitivity and specificity in neonates of 89% (80–94%) and 60% (54–66%), respectively, when a 80 pg/mL threshold is used in neonates [23].

#### 3.1.2. The Use of BDG in Children with Cancer or Post HSCT

For BDG used in paediatric cancer patients, the search retrieved 88 publications. Among those, seven studies analysed the value of BDG in the early detection of invasive fungal disease in immunocompromised children, which included between 24 and 471 patients (median, 125 patients) [24,25,26,27,28,29,30]. An additional study investigating BDG in children with cancer was published in Chinese and was not included in the analysis [31]. Whereas some studies included patients at high risk of invasive fungal disease only, e.g., patients treated for AML or undergoing allogeneic hematopoietic cell transplantation (HCT) [24,27,29], other studies included also patients treated for solid tumours or undergoing abdominal surgery who are generally considered to be at low risk of invasive fungal disease [25,28]. All studies used a cut-off of 80 pg/mL. The prevalence of invasive fungal disease in the study population ranged between 3% and 17.6% in the five studies which used BDG for screening [24,26,27,29,30]. One study used BDG as a diagnostic tool in children with signs and symptoms suggestive of invasive fungal disease [25], and one study included both strategies in the analysis [28]. In the analysis of studies investigating BDG for screening, the range (median) of specificity, sensitivity, positive and negative predictive value (PPV, NPV) was as follows: 29–95% (55%), 0–100% (75%), 0–25% (17%), and 84–100% (95%), respectively. In the study in which BDG was used as diagnostic tool, specificity, sensitivity, PPV and NPV were 47%, 80%, 69%, and 71%, respectively.

Our search aimed to include primary immunodeficiencies but this did not retrieve any references.

### 3.2. Survey Results

**Figure 1:**
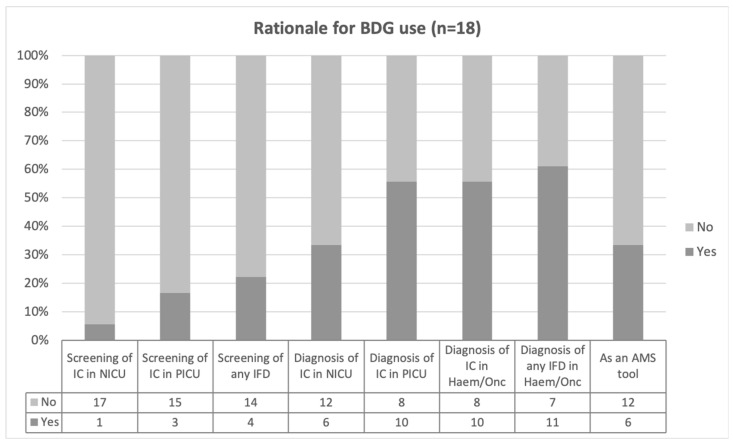
Proportion of positive responses to the different rationales for using Beta-D-Glucan (BDG) from a total of 18 positive answers. IC, invasive candidiasis; NICU, neonatal intensive care unit; PICU, paediatric intensive care unit; IFD, invasive fungal disease; AMS, antimicrobial stewardship.

The survey was completed by 54 responders; 66.7% (36/54) from 17 European countries, 22.2% (12/54) from an American or Asiatic country, and six (11.1%) which did not specify the country. Within Europe, half of the responders (18/36) referred to the use of BDG in their routine clinical practice, with half of them having the test in-house (9/18). The rationale behind the use of the BDG test is summarised in Figure 1. BDG was reported as being more frequently used as diagnostic rather than as a screening tool. The diagnostic use was limited in neonates (6/18, 33.3%), whereas the haemato-oncology and paediatric intensive care unit (PICU) had each 11/18 (61.1%), and 10/18 (55.6%) positive responses. BDG was reported as an antimicrobial stewardship (AMS) tool in one third of the participants (6/18). More than half of the responders (10/18, 55.6%) using BDG in their clinical practice did not know the cut-off value of positivity whereas four (4/18, 22.2%) used a cut-off different to 80 pg/ml, the defined cut-off by the manufacturer. For those not using the BDG assay, 11/18 (61.1%) reported a lack of access as the main reason. Compared to BDG, there were more responders reporting the use of GM (29/36, 80.6%). From these, it was more common to have in-house access to this test (18/29, 62.1%).

## 4. Discussion

The clinical signs and symptoms of invasive fungal disease are often non-specific in premature neonates and in immunocompromised and/or critically ill children, which often make an early diagnosis difficult. On the other hand, studies in adults demonstrate that early diagnosis and treatment is associated with better outcomes [32]. Standard diagnostic procedures include culture, microscopy and histology, and do not differ between children and adults. All these methods, however, have limited sensitivity, and unfortunately, the results of culture-based techniques are not readily available [6]. Imaging is one cornerstone in the diagnostic strategies of invasive mould disease, but imaging findings can neither prove invasive mould disease nor differentiate between different fungal pathogens. In addition, the benefit and potential harm of imaging studies have to be weighed cautiously, as they might be associated with radiation exposure (e.g., computerized tomography (CT) scan) or with the need of anaesthesia, in particular in younger children. There is growing interest in non-culture based assays such as PCR, GM or BDG. These assays require less sample volume, which is important in particular in the neonatal setting, and have a fast turn-around time compared to culture-based assays, which allows earlier initiation of specific antifungal therapy.

For a long time, the value of PCR assays in the diagnosis of invasive fungal disease was unclear, as assays differed in the gene sequence detected (e.g., assays were specific for *Aspergillus fumigatus*, for *Aspergillus* species, or were “pan-fungal” tests). In addition, the PCR assays, which were mostly used as in-house tests, differed regarding sample origin (e.g., full blood, serum or plasma) and methodology. However, based on the work of the International Society for Human and Animal Mycology (ISHAM) working group fungal PCR initiative, it became clear that the test results of PCR assays, which meet certain standards, are comparable [33,34,35]. The encouraging data resulted in the inclusion of PCR as a mycological criterion in the updated revised definitions of invasive fungal disease by the European Organisation for Research and Treatment of Cancer/invasive Fungal Infections Cooperative Group and the Mycosis Study Group (EORTC/MSG) consensus group [36]. Although paediatric data are limited, the paediatric specific guidelines given by the ECIL-8 panel recommend the detection of fungal nucleic acids in broncho-alveolar lavage (BAL) fluid, in the cerebrospinal fluid (CSF), body fluids and tissue specimens whenever these specimens are obtained [12]. Notably, PCR assays should preferentially be performed in a reference laboratory. In order to further improve molecular diagnostics of invasive fungal disease, next generation sequencing (NGS) are promising techniques, and serum microbial cell-free DNA sequencing revealed high specificity, but moderate sensitivity in adult HSCT recipients with pulmonary aspergillosis. It has to be mentioned that other non-culture based techniques for the early diagnosis of invasive fungal disease are also being evaluated, such as the assessment of the fungal-induced release of T-cellular signature cytokines [37]. Although these techniques are promising, they have not been extensively tested in the setting of immunocompromised patients, and data in children and adolescents are lacking to date.

Multiple studies in children, adolescents and adults have demonstrated the usefulness of GM, which is included in the updated revised consensus definitions of invasive fungal disease by the EORTC/MSG. The detection of GM, which is a fungal cell wall antigen, in blood, BAL or the CSF may indicate invasive aspergillosis. False-positive results can be caused by a variety of reasons, such as the concomitant administration of some beta-lactam antibiotics, or by cross-reactivity with non-aspergillus fungal species such as *Penicillium marneffei*, *Histoplasma capsulatum* and *Cryptococcus neoformans* [6]. On the other hand, the use of mould-active prophylaxis increases the rate of false-negative findings, which explains the fact that GM screening twice weekly is recommended for patients at high risk for invasive aspergillosis not receiving mould-active prophylaxis, but not for those receiving prophylaxis [12]. Although the performance of GM in children is similar to that in adults [38], the performance of modified tests such as the lateral flow device (LFD), which can be used as a point of care diagnostic assay, has not been fully evaluated in children [39].

1,3-beta-D-glucan, is a major cell wall component of most fungal species with the exception of Mucormycetes and *Cryptococcus* spp. [40]. Therefore, compared to GM, BDG may be detected not only in invasive aspergillosis, but also in invasive candidiasis, which is particularly important in the neonatal setting. Four commercial BDG antigen assays are available (Fungitell^®^, Fungitec-G^®^, Wako^®^ and Maruha^®^), and it is important to note that the cut-off levels range from 11 pg/mL to 80 pg/mL, depending on the assay. Whereas the last three assays are available in Japan only, the Fungitell^®^ assay is available in the US and Europe. Suitable specimens for BDG testing are blood and the CSF.

In contrast to adults, the clinical evidence supporting the use of BDG in children is still scarce and there is significant variability depending on study population, study design, rationale and setting of use. This is clearly demonstrated by the results of our survey, which showed that the use of BDG in the paediatric population is not uniformly extended in Europe, with limitations in access to testing and a lack of a clear threshold for positivity.

Overall, the studies we included in our review demonstrate a relatively low specificity and PPV, whereas the NPV is considerably high. Unfortunately, data from the neonatal and paediatric setting are limited and heterogeneous, which might be explained in part by factors such as study design, heterogeneous patient populations (mixed risk profiles for invasive fungal disease), colonisation status and inconsistencies in the use of antifungal prophylaxis. All these factors lead to different pre-test probabilities, which affect the performance of the test. Three additional issues have to be emphasized in more detail.

First, it remains unclear in some of the studies whether a positive BDG assay was defined by the results of one single or two consecutive BDG assessments. Compared to different diagnostic windows, the impact of the different definitions on sensitivity and specificity seems to be smaller, as reported by two studies [26,27].

Second, it has been reported that BDG levels are higher in healthy children compared to adults [13]; and in healthy neonates compared to older children [23]. Therefore, an age-dependent threshold might be necessary; as false positivity of BDG has been associated with younger age [26], as well as the need for a higher cut-off for positivity to result in similar sensitivity for the neonatal age [14]. Unfortunately, to date, no specific cut-off level has shown agreement throughout the different studies in this age group [14,15,16,17,28]. On the other hand, in severe burn patients, a lower cut-off was more accurate in detecting candidaemia [41]. These data support that the optimal cut-off of BDG likely needs to be defined for different age groups and possibly for different clinical settings, which should be stated as a major research gap.

The clinical utility of a test is reflected by parameters such as sensitivity, specificity, PPV and NPV, respectively. Whereas a meta-analysis of the value of BDG in adult patients reports an overall sensitivity of 75 to 83% and specificity of 63 to 87%, respectively [42], data in the paediatric setting are less clear. Our data on BDG in children reveal a wide range of specificities with a median of 73%. Regardless of age, there is a long list of reasons for false-positive results, which includes the receipt of intravenous immunoglobulin (IVIG), albumin, and blood products (e.g., packed red blood cells [PRBCs] or fresh frozen plasma [FFP]), intravenous amoxicillin/clavulanate, colistin, cefazolin, trimethoprim-sulfamethoxazole, cefotaxime, cefepime, PEG-asparaginase and defibrotide [42]. In addition, Gram-negative and Gram-positive bacteraemia, fungal colonisation, mucositis or other disruptions of gastrointestinal integrity, haemodialysis membranes, surgical gauze containing glucan or the sampling method may also result in false-positive results [42]. Other factors, such as antifungal exposure or high concentrations of bilirubin or triglycerides have been reported to reduce BDG levels [19,43]. The relatively low specificity, however, may result in the over-diagnosis of invasive fungal diseases, which leads to unnecessary antifungal treatment. The poor specificity is particularly problematic in immunocompromised patients, in whom it is extremely difficult to withhold antimicrobial compounds if a serum parameter may suggest an infection, which is similar in high-risk neonates. Notably, the ECIL-3 guidelines for adult high-risk patients recommend the use of BDG results for clinical management decisions in conjunction with clinical, microbiological and radiological findings [8]. Although the sensitivity of BDG exceeded 90% in two studies [27,29], it is important to note that a negative BDG does not exclude mucormycosis, which is a rare but increasing clinical problem [44].

These uncertainties are reflected in the fact that in the 2019 revised and updated definitions for invasive fungal disease by the EORTC/MSG, BDG as a microbiological criterion for the diagnosis of a probable invasive fungal disease was removed [36]. In the setting of patients in the intensive care unit (ICU), current guidelines hesitate to give concrete recommendations about antifungal therapy based on BDG results only, and an on-going randomised controlled trial (CandiSep, https://clinicaltrials.gov/ct2/show/NCT02734550, accessed on 14 November 2022) is looking to provide additional support for the role of BDG and the potential impact on time to empirical treatment and patient survival [45].

## 5. Conclusions

In conclusion, the early diagnosis of invasive fungal disease is difficult, and there is growing interest in the development of non-culture based diagnostic tests such as BDG. Although preliminary data on BDG in the paediatric setting are promising, there are a number of unanswered questions which currently limit the routine use of serum BDG in children and adolescents (Table 1). This problem was also reflected in the results of our survey, which showed diversity in access and use. Further studies are urgently warranted in order to optimize the performance of BDG in neonates and immunocompromised children which ultimately may help to decrease the morbidity and mortality of invasive fungal disease.

## Figures and Tables

**Table 1:** Challenges and open questions to be addressed in future paediatric studies.

Comparable Study Designs	Analyses Needed
Patient populations	Calculating the optimal cut-off for the different age groups
Strategy: screening/diagnostics Definition of diagnostic window	Optimal definition of BDG positivity

## Data Availability

Not applicable.

## Supplementary Materials

The following supporting information can be downloaded at: https://www.mdpi.com/article/10.3390/jof8121262/s1.
