# Supplementary material for: Serum Beta-D-Glucan in the Diagnosis of Invasive Fungal Disease in Neonates, Children and Adolescents: A Critical Analysis of Current Data
Source: J Fungi (Basel). 2022 Nov 30;8(12):1262. doi: 10.3390/jof8121262 (PMC9783846; doi:10.3390/jof8121262)
Supplement: Supplementary file 1 [file jof-08-01262-s001.zip › jof-2014477-supplementary.pdf]

ß-D-glucan and other fungal markers use in Neonates and Children in Europe

Thank you for completing the short survey below.

Country	◯ Austria<br>◯ Belgium<br>◯ Bulgaria<br>◯ Croatia<br>◯ Republic of Cyprus<br>◯ Czech Republic<br>◯ Denmark<br>◯ Estonia<br>◯ Finland<br>◯ France<br>◯ Germany<br>◯ Greece<br>◯ Hungary<br>◯ Ireland<br>◯ Italy<br>◯ Latvia<br>◯ Lithuania<br>◯ Luxembourg<br>◯ Malta<br>◯ Netherlands<br>◯ Poland<br>◯ Portugal<br>◯ Romania<br>◯ Switzerland<br>◯ Slovakia<br>◯ Slovenia<br>◯ Spain<br>◯ Sweden<br>◯ Turkey<br>◯ United Kingdom<br>◯ Other
Other country, please specify:	__________________________________
Do you use ß-D-glucan in your routine clinical practice in paediatrics?	◯ Yes<br>◯ No
What is the rationale for using the ß-D-glucan test?	☐ Screening of invasive candidiasis in NICU<br>☐ Screening of invasive candidiasis in PICU<br>☐ Screening for any invasive fungal disease regardless setting<br>☐ As a diagnostic tool of invasive candidiasis in NICU<br>☐ As a diagnostic tool of invasive candidiasis in PICU<br>☐ As a diagnostic tool of invasive candidiasis in haemato-oncology patients<br>☐ As a diagnostic tool of any invasive fungal disease, in haemato-oncology patients<br>☐ As an antimicrobial stewardship tool<br>(Please, tick all which apply. NICU, Neonatal Intensive Care Unit; PICU: Paediatric Intensive Care Unit.)
Do you have access to in-house ß-D-glucan testing or is the test sent to a different laboratory?	◯ Test is done at my institution<br>◯ Test is done at a different laboratory

When using ß-D-glucan, which is the cut-off that you consider as positive?	◯ Positive when > 80 pg/mL<br>◯ Other<br>◯ Not known
Other level (pg/mL)	__________________________________
Is ß-D-glucan available in the microbiology laboratory at your institution?	◯ Yes<br>◯ No
Then, why not?	__________________________________
Do you use any other fungal markers in your routine clinical practice?	☐ Galactomannan (GM)<br>☐ Others<br>(Please, tick all which apply)
What is the rationale for using GM?	☐ As a screening of invasive aspergillosis<br>☐ As a diagnostic tool of invasive aspergillosis<br>☐ As an antimicrobial stewardship tool<br>(Please, tick all which apply.)
Do you have access to in-house GM testing or is the test sent to a different laboratory?	◯ Test is done at my institution<br>◯ Test is done at a different laboratory
Which samples would you normally test for GM?	☐ Serum<br>☐ Bronchoalveolar lavage (BAL) fluid<br>☐ Cerebrospinal fluid (CSF)<br>(Please, tick all which apply)
Which value do you consider positive for GM in a serum sample?	◯ ≥ 0.5<br>◯ ≥ 0.7<br>◯ ≥ 1.0<br>◯ Other<br>◯ Not known
Which value do you consider positive for GM in a BAL sample?	◯ ≥ 0.5<br>◯ ≥ 0.7<br>◯ ≥ 1.0<br>◯ Other<br>◯ Not known
Which value do you consider positive for GM in a CSF sample?	◯ ≥ 0.5<br>◯ ≥ 0.7<br>◯ ≥ 1.0<br>◯ Other<br>◯ Not known
Which other markers?	__________________________________
